# Ns1 Is a Key Protein in the Vaccine Composition to Protect Ifnar(−/−) Mice against Infection with Multiple Serotypes of African Horse Sickness Virus

**DOI:** 10.1371/journal.pone.0070197

**Published:** 2013-07-23

**Authors:** Francisco de la Poza, Eva Calvo-Pinilla, Elena López-Gil, Alejandro Marín-López, Francisco Mateos, Javier Castillo-Olivares, Gema Lorenzo, Javier Ortego

**Affiliations:** 1 Centro de Investigación en Sanidad Animal, INIA, Valdeolmos, Madrid, Spain; 2 The Pirbright Institute, Pirbright, Woking, Surrey, United Kingdom; University of Melbourne, Australia

## Abstract

African horse sickness virus (AHSV) belongs to the genus *Orbivirus*. We have now engineered naked DNAs and recombinant modified vaccinia virus Ankara (rMVA) expressing VP2 and NS1 proteins from AHSV-4. IFNAR^(−/−)^ mice inoculated with DNA/rMVA-VP2,-NS1 from AHSV-4 in an heterologous prime-boost vaccination strategy generated significant levels of neutralizing antibodies specific of AHSV-4. In addition, vaccination stimulated specific T cell responses against the virus. The vaccine elicited partial protection against an homologous AHSV-4 infection and induced cross-protection against the heterologous AHSV-9. Similarly, IFNAR^(−/−)^ mice vaccinated with an homologous prime-boost strategy with rMVA-VP2-NS1 from AHSV-4 developed neutralizing antibodies and protective immunity against AHSV-4. Furthermore, the levels of immunity were very high since none of vaccinated animals presented viraemia when they were challenged against the homologous AHSV-4 and very low levels when they were challenged against the heterologous virus AHSV-9. These data suggest that the immunization with rMVA/rMVA was more efficient in protection against a virulent challenge with AHSV-4 and both strategies, DNA/rMVA and rMVA/rMVA, protected against the infection with AHSV-9. The inclusion of the protein NS1 in the vaccine formulations targeting AHSV generates promising multiserotype vaccines.

## Introduction

African horse sickness virus (AHSV) is an *Orbivirus* of the family *Reoviridae* that causes a severe disease in equids. In susceptible horses the mortality could reach 90%. Although African horse sickness (AHS) is mostly confined to sub-Saharan Africa, there are sporadic outbreaks in North Africa, Pakistan, India, Portugal and Spain [1]. Nine serotypes of the virus, AHSV-1 to AHSV-9, have been described [2], [3]. The genus *Orbivirus* also includes bluetongue virus (BTV) and epizootic haemorrhagic disease virus (EHDV), which have similar morphological and biochemical properties but affect different hosts. Moreover, AHSV is transmitted by *Culicoides* midges [1], [4], the same insect vectors as those that transmit BTV. Since 2008 there has been a dramatic northward spread of BTV in Europe related with the extension of the insect’s habitat due to climate change. Therefore the presence of insect vectors in Europe increases the probability that outbreaks of AHS may follow [5].

AHSV is a non-enveloped, icosahedral symmetric virus with ten linear segments of double-stranded RNA. AHSV virions are composed of seven structural proteins (VP1-VP7) arranged as three concentric layers surrounding the genome [6]. VP2 and VP5 are the outer capsid proteins, while the core surface layer is composed of VP7 and VP3 forms the inner capsid of the virion. Proteins VP1, VP4 and VP6 constitute core associated transcriptase complexes. There are four nonstructural proteins (NS1, NS2, NS3/3A, and NS4), involved in virus replication, morphogenesis and release from the infected cell [7], [8], [9].

Vaccination with a live-attenuated polyvalent AHSV vaccine is used to control the disease in Africa. However, this type of vaccine causes viraemia in the host and therefore has the potential to be acquired by the vector and transmitted in the field. In addition, a recent study showed that horses immunized against AHSV can be infected both clinically and subclinically with AHSV following natural infection in field conditions. Indeed, the level of viraemia observed in subclinically infected horses might be sufficient to infect midges with AHSV [10]. These attenuated vaccines have other disadvantages, such as the possible exchange of genome segments with field strains and the impossibility to distinguish (naturally) infected and vaccinated animals (‘DIVA’),

Recently, a recombinant vaccine based on MVA expressing VP2 protein (rMVA-VP2) showed its efficacy eliciting neutralizing antibodies in ponies [11] and protection in mice against homologous challenge [12]. VP2 contains the major neutralizing epitopes; however, they are serotype-specific [13], [14]. Other recombinant vaccines, expressing VP2 [15] or VP2/VP5 [16] protected against homologous challenge, however no previous heterotypic vaccination studies have been described.

Sequences of the NS1 gene are highly conserved between the different serotypes of AHSV [7]. Although little is known about the role of AHSV NS1 in host immune response, multiple CTL epitopes are present on non-structural NS1 protein of BTV [17], [18]. Moreover, previous studies from our group demonstrated that the inclusion of NS1 in a vaccination strategy based on DNA/MVA expressing VP2 and VP7 proteins enhanced cross-protection against heterologous serotypes of BTV [19]. Therefore, we considered of interest to determine whether AHSV NS1 might be similarly able to enhance the level of cross-protection in a vaccination strategy against heterologous challenge.

Interferon alpha/beta receptor knockout (IFNAR^(−/−)^) mice have been characterized as a suitable animal model for AHSV, BTV and EHDV, since these mice are able to support the *in vivo* growth of these orbiviruses and they show viraemia and clinical signs. In addition, our previous results [12], [19], [20], [21] and those from other groups [22], [23], [24], [25], [26] have shown that the IFNAR^(−/−)^ infection model is useful for the definition of effective recombinant vaccine candidates against several viruses.

In the present study, we have determined the protection of IFNAR ^(−/−)^ mice vaccinated with DNA/rMVA or rMVA/rMVA expressing VP2 and NS1 proteins from AHSV-4 against homologous or heterologous challenge (AHSV-9). As well the immune response elicited by these vaccination regimes was analyzed in the mouse model.

## Materials and Methods

### Virus and Cells

Baby hamster kidney (BHK-21) (ATCC, Cat. No. CCL-10), chicken embryo fibroblast (DF-1) (ATCC, Cat. No. CRL-12203), and Vero (ATCC, Cat. No. CCL-81) cells were grown in Dulbeccós modified Eaglés medium (DMEM) supplemented with 2 mM glutamine and 10% fetal calf serum (FCS). AHSV serotype 4 (Madrid-87) (AHSV-4) and AHSV serotype 9 (PAKrrah/09) (AHSV-9) were used in the experiments. Standard virus titrations were performed in Vero cells. Virus stocks were generated by infection of confluent Vero cells using a multiplicity of infection (MOI) of 1. At 48 hours post-infection (h.p.i.), or when total cytopathic effect (CPE) was visible, the cells and supernatants were harvested and centrifuged. The virus were released from the cells by three freeze and thaw cycles. Modified vaccinia virus Ankara (MVA) was growth and titered in DF-1 cells using a MOI of 1.

### Mice

IFN α/βR^o/o^ IFNAR^(−/−)^ mice, on a 129 background were purchased from B&K Universal Ltd UK. Eight-week old male mice were used throughout. Mice were maintained under pathogen-free conditions and allowed to acclimatize to the biosafety level 3 (BSL3) animal facility at the Centro de Investigación en Sanidad Animal, INIA, Madrid, for 1 week before use in our experiments. All experiments with live animals were performed under the guidelines of the European Community (86/609) and were approved by the ethical review committee at the Centro de Investigación en Sanidad Animal of the Instituto Nacional de Investigaciones Agrarias (Permit number: CEEA 2010-034). All efforts were made to minimize suffering.

#### Cloning of VP2 and NS1 AHSV-4 genes and generation of recombinant MVAs

Segments 2 and 5 corresponding to VP2 and NS1 respectively were amplified from AHSV-4. To generate pcDNA3-VP2, the restriction site *SmaI* was introduced into the 5′ and 3′ ends of the PCR product. The oligonucleotide primers AHSV-4-VP2-SmaI (1) VS (5′-CGCCCGGGATGGCGTCCGAGTTTGGAATATTG -3′), and AHSV-4-VP2-SmaI (3183) RS (5′-CGCCCGGGCTATTCCGTTTTTGCGAGTAACTTCG -3′, *Sma I* site underlined), were used to generate a PCR product comprising AHSV-4 gene VP2.

To generate pcDNA3-NS1 and the MVA transfer plasmid pSC11-NS1, the restriction site *SmaI* was introduced into the 5′ and 3′ ends of the PCR product. The oligonucleotide primers AHSV-4-NS1-SmaI (1) VS (5′-CGCCCG GGATGGATAGGTTCTTGACTTATTTC -3′), and AHSV-4-NS1-SmaI (1647) RS (5′-CGCCCGGGCTAATTATGCATGAAATCAAAGGG -3′, *Sma I* site underlined), were used to generate a PCR product comprising AHSV-4 gene NS1.

PCR products were digested with *SmaI* and cloned into the *SmaI* digested pSC11 to generate pSC11-NS1plasmid or into *EcoRV* digested pcDNA3 plasmid to generate, pcDNA-3-NS1 and pcDNA3-VP2. The generated plasmids were sequenced to analyze the right orientation of the cloned VP2 and NS1 genes.

The MVA transfer plasmid pSC11-NS1 contained the NS1 AHSV gene inserted into the thymidine kinase site of MVA and under the control of the vaccinia virus (VV) early/late promoter p7.5. Recombinant MVA (rMVA) were prepared by infecting DF-1 cells with MVA at a multiplicity of infection of 1 (MOI = 1) and transfecting them with the transfer plasmid pSC11-NS1. Cell cultures were harvested at 48 h.p.i., and the rMVA were selected after plaque assay by the addition of X-Gal to the agar overlay. rMVA was cloned four times by plaque isolation assay and the purity of the rMVA-NS1 analyzed by PCR by using the oligonucleotide primers used to amplify the BTV gene and described above. The rMVA-VP2 has been previously described [11].

### Immunofluorescence

Cells were plated on glass coverslips and they were infected or transfected. Infections were performed at an MOI of 1 PFU/cell at 37°C in DMEM containing 2% FCS. Free viruses were removed after 90 minutes and the cells were maintained in DMEM 2% FCS. Transfections were performed with lipofectamine 2000 (Invitrogen) with 4 µg of DNA, according to the method recommended by the manufacturer. At the indicated times, the cells were washed with PBS and fixed by addition of 4% paraformaldehyde for 30 min at room temperature. Cells were incubated with a PBS-FCS 20% diluent containing 0.2% Saponin (Superfos-Biosector, Vedback, Denmark) for 1 hour at room temperature. Mouse polyclonal antibody specific for AHSV-4 was allowed to adsorb for 90 min at room temperature, and washed three times with PBS-FCS 2%. Cells were then incubated for 30 min at room temperature with an anti-mouse secondary antibody conjugated to Alexa 594. The coverslips were washed five times with PBS-FCS 2%, mounted on glass slides, and analyzed with a Olympus CKX41 microscope.

#### Prime-boost immunization and challenge with AHSV in IFNAR^(−/−)^ mice

Groups of five IFNAR^(−/−)^ mice were immunized by homologous or heterologous prime-boost vaccination with rMVA or DNAs and rMVAs respectively, expressing AHSV-4 proteins or pcDNA3 and MVA (non-immunized mice), administered 3 weeks apart. A suspension of 50 µg of each pcDNA3 construct was administered intramuscularly and 10^7^ PFUs of each rMVA construct were inoculated intraperitoneally. Two weeks after immunization all mice were subcutaneously inoculated with 10^6^ PFUs of AHSV-4 or AHSV-9. Mice were bled before each immunization and virus challenged. Sera were tested for AHSV-4 and AHSV-9 neutralizing antibodies by standard Virus Neutralization Test (VNT).

### Detection of AHSV-4 and AHSV-9 in Blood

Whole blood was collected in EDTA from all animals at regular intervals after inoculation. The viruses were released from whole blood by three freeze/thaw cycles. The amount of infectious virus was measured by plaque assay on Vero cells.

### AHSV-4 and AHSV-9 Neutralizing Antibody Detection in Immunized Mice by VNT

The VNT was used to determine neutralizing antibody titers against AHSV-4 or AHSV-9. For plaque reduction assays, 2 fold dilutions of sera were mixed with 100 PFU of AHSV-4 or AHSV-9, incubated for 1 hour at 37°C and then plated into monolayers of Vero cells. After 1 hour, agar overlays were added and the plates were incubated for 5 days. The titer was determined as the highest dilution that reduced the number of plaques by 50%.

### UV Inactivation of AHSV

Extracts of Vero cells infected with AHSV-4 or AHSV-9 were exposed to UV light (400 _J/cm2) for 30 min [27]. The effectiveness of this treatment at inactivating the virus was confirmed by plaque assay. Confluent cultures of Vero cells were infected with the UV-inactivated virus as previously described by using the intact virus.

### IFN-γ ELISPOT Assays

ELISPOT assays were performed with Mouse IFN gamma ELISPOT Ready-SET-Go (eBioscience), according to the method recommended by the manufacturer. A total of 4×10^5^ splenocytes were added to the well and stimulated with 10^4^ PFUs of UV inactivated virus. Plates were incubated at 37°C and 5% CO_2_ for 18–20 hours. As a positive control, PHA was used. Plates were scanned on an ImmunoSpot reader (Cellular Technology Ltd.). Specific spots were counted using the Immuno-Spot software. The threshold values to consider a positive response by ELISPOT was that the number of specific spots/well had to be at least 2 times the average values found in negative control wells of each group, and that after subtraction of background values, responses had to be higher than 20 SFC/million splenocytes.

### Detection of Epitope-specific CD4^+^ and CD8^+^ T-cell Responses by Intra-cellular Cytokine Staining (ICCS)

Mice immunized with rMVA or DNA were sacrificed at 14 days post-booster and their spleens were harvested for analysis by ICCS assay. A total of 10^6^ splenocytes were stimulated with 10^4^ PFUs of inactivated virus per well or left untreated during 18 hours in RPMI 1640 supplemented with 10% FCS and containing brefeldin A (5 µg/ml) to increase the accumulation of gamma interferon (IFN-γ) in the responding cells. After stimulation, cells were washed, stained for the surface markers, fixed, permeabilized and stained intracellularly using the appropriate fluorochromes. To analyze the adaptive immune responses, the following fluorochrome-conjugated antibodies were used: CD4-FITC, CD8-PerCP and IFNγ-PE. All antibodies were from BD Biosciences. Data were acquired by FACS analysis on a FACSscalibur (Becton Dickinson) and were analyzed with CellQuest Pro software.

## Results

### Evaluation of Protein Expression in Cells Tranfected with cDNAs or Infected with Recombinant MVAs Expressing VP2 and NS1 Proteins from AHSV-4

**Figure 1:**
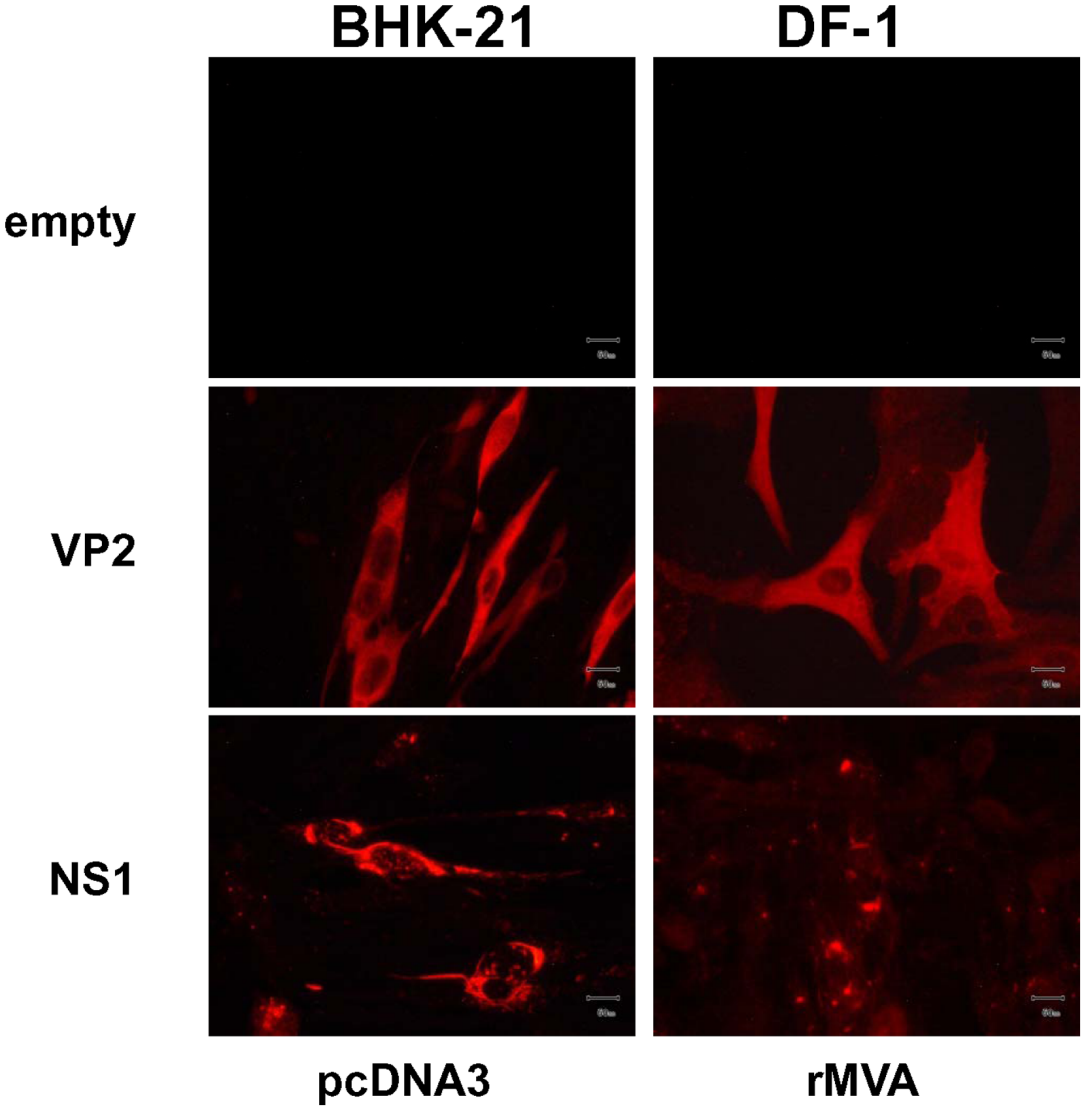
Expression of the VP2 and NS1 AHSV-4 proteins. Immunofluorescence microscopy using a mouse polyclonal antibody against AHSV-4 in BHK-21 cells transfected with pcDNA3, pcDNA3-VP2 or pcDNA3-NS1 plasmids and in DF-1 cells infected with rMVA, rMVA-VP2 or rMVA-NS1. Scale bar, 50 µm.

In order to evaluate the expression of the AHSV-4 recombinant VP2 and NS1 proteins from pcDNA3 and rMVAs vectors in transfected BHK-21 and infected DF-1 cells, respectively, transient expression studies using immunofluorescence microscopy (IFA) were performed. Labeling was observed on BHK-21 cells transfected with pcDNA3-VP2 and pcDNA3-NS1 by using a serum of mice infected with serotype 4 of AHSV, but not on cells transfected with the control plasmid pcDNA3 (Fig. 1). Expression of VP2 and NS1 proteins was also observed in DF-1 cells infected with rMVA-VP2, and rMVA-NS1, respectively, but not in control MVA infected cells (Fig. 1). These data confirmed the efficient expression of the proteins from AHSV-4 cloned in the DNA and MVA vaccine vectors used for immunization of IFNAR^(−/−)^ mice.

### Heterologous Prime-boost Immunization with pcDNA3-VP2/pcDNA3-NS1 and rMVA-VP2/rMVA-NS1 Partially Protects IFNAR^(−/−)^ Mice against Homologous AHSV-4 and Heterologous AHSV-9 Infection

Adult IFNAR^(−/−)^ mice were immunized first with pcDNA3-VP2 and/or pcDNA3-NS1 by intramuscular route. After three weeks, mice were inoculated intraperitoneally with a booster of rMVA-VP2 and/or rMVA-NS1. Two weeks after the second immunization, immunized and control IFNAR^(−/−)^ mice were challenged subcutaneously with 10^6^ PFUs of AHSV-4 or 10^6^ PFUs of AHSV-9. These doses of AHSV-4 have been previously described to induce clinical signs, viraemia and significant levels of lethality [12]. All mice infected with AHSV-4 presented clinical signs, which were more evident in non-immunized mice and animals immunized with NS1 (Table 1). These included presentation of rough hair coat, a hunched posture and reduction of mobility, which in some cases led to lethargy. Lacrimation and swelling of the eyelids was also observed in non-immunized or NS1 immunized animals. Non-immunized mice infected with AHSV-9 presented a milder clinical syndrome characterised by reduction of mobility and eye swelling. In contrast, the groups of animals immunized with NS1, VP2 or both antigens remained healthy to the end of the study (Table 1).

**Table 1:** Post-challenge sickness score in mice vaccinated with indicated vaccine regimens.

			Days post-challenge			
Immunization	Challenge	Group	3	5	7	10
**DNA/rMVA**	**AHSV-4**	**Non immun.**	2.4±0.54	4.2±0.83	4.2±0.83	2.75±0.95
		**VP2**	0	2.8±0.44	0	0
		**NS1**	1.4±0.54	3.8±0.83	3.6±0.89	3±1.22
		**VP2+NS1**	2±0.70	2.6±0.54	3,8±1,3	46±0.57
	**AHSV-9**	**Non immun.**	0	3.4±0.61	1.4±0.7	0
		**VP2**	0	0	0	0
		**NS1**	0	0	0	0
		**VP2+NS1**	0	0	0	0
**rMVA/rMVA**	**AHSV-4**	**Non immun.**	1.6±0.54	4.8±0.44	4.3±0.57	0
		**VP2**	0	0	0	0
		**NS1**	2.4±0.54	3.4±0.54	4.2±0.09	0
		**VP2+NS1**	0	0	0	0
	**AHSV-9**	**Non immun.**	0	3.2±0.44	1.25±0.5	0
		**VP2**	0	0	0	0
		**NS1**	0	0	0	0
		**VP2+NS1**	0	0	0	0

Mice were evaluated and scored for individual symptoms. Rough hair (absent = 0, present = 1), activity (normal = 0, reduced = 1, severely reduced = 2), hunched (absent = 0, present = 1), eye swelling (absent = 0, present = 1). The final score was the addition of each individual score. The minimum score was 0 for healthy and 1–5 depending upon the severity. Each score represents the mean values of six animals and the standard deviation.

**Figure 2:**
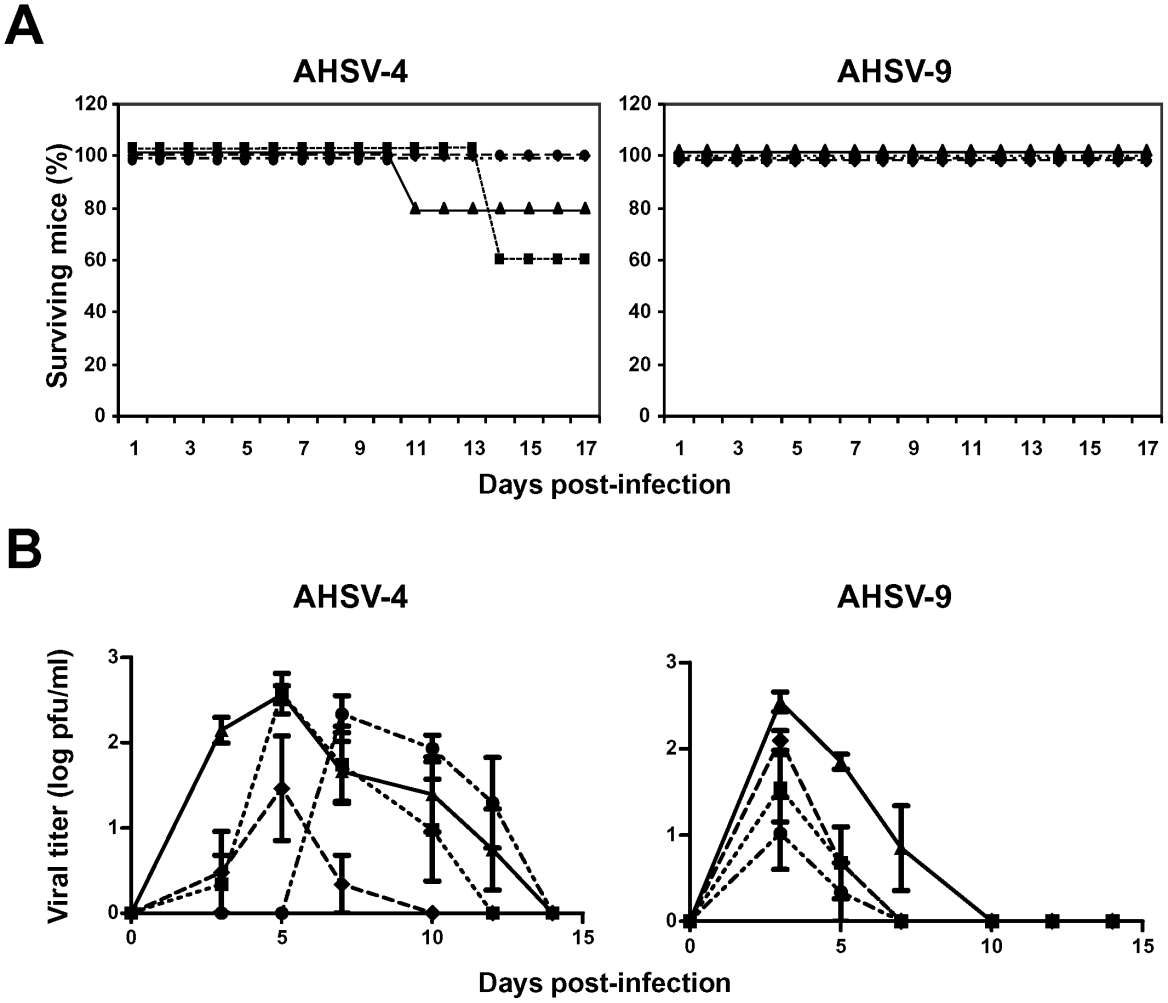
Protection of DNA/rMVA vaccinated IFNAR^(−/−)^ mice against an AHSV-4 or AHSV-9 challenge. Mice (8 weeks old, 6 per group) were immunized twice by heterologous prime-boost vaccination with pcDNA3 and rMVA expressing VP2 (**♦**), NS1 (▪), or VP2/NS1 (•), AHSV-4 proteins or pcDNA3 and MVA **(**▴**)** (non-immunized mice), administered 3 weeks apart. Two weeks after immunization all mice were subcutaneously inoculated with 10^6^ PFUs of AHSV-4 or AHSV-9. (A) Survival rates of immunized and non-immunized IFNAR^(−/−)^ mice after inoculation with AHSV-4 or AHSV-9. The mice were observed every 24 h for 17 days. (B) Titers of AHSV-4 or AHSV-9 recovered in blood of immunized and non-immunized IFNAR^(−/−)^ mice after challenge. Virus was extracted from blood and determined as described in Materials and Methods. Each point represents the mean values of the viral titer of six animals, and standard deviations are shown as error bars.

While 20% of non-immunized and 40% of NS1 immunized animals died, 100% of the animals immunized with VP2 or VP2-NS1 survived to the challenge with AHSV-4. All the animals, immunized or not immunized, infected with AHSV-9 survived to the challenge (Fig. 2A).

The titers of infectious virus recovered in the blood after challenge with AHSV-4 or AHSV-9 were determined in immunized and non-immunized IFNAR^(−/−)^ mice by plaque assay. In the animals infected with AHSV-4, titers up to 5×10^2^ PFU/ml were observed at 5 days post-challenge in non-immunized and NS1 immunized animals. Lower level of viraemia was detected in the animals immunized with VP2 and a delay in the appearance of infective virus in blood in the animals immunized with VP2/NS1. In the groups of animals challenged with AHSV-9, titers up to 4×10^2^ PFU/ml were detected at 3 days post-challenge in non-immunized mice. Titers up to 10^2^ and 0.7×10^2^ PFU/ml were detected in the animals immunized with VP2 and NS1, respectively, at the same day post-challenge. Mice immunized with VP2/NS1 showed lower viral titers than the other groups challenged with AHSV-9 (Fig. 2B).

These data suggested that the immunization of mice with VP2 partially protected the animals against an homologous challenge with AHSV-4. Furthermore, VP2/NS1 of AHSV-4 induced protection against serotypes 4 and 9 of AHSV upon heterologous prime-boost immunization using naked DNA and rMVA as vaccine vectors.

### Homologous Prime-boost Immunization with rMVA-VP2/rMVA-NS1 Protects IFNAR^(−/−)^ Mice against Homologous AHSV-4 and Heterologous AHSV-9 Infection

**Figure 3:**
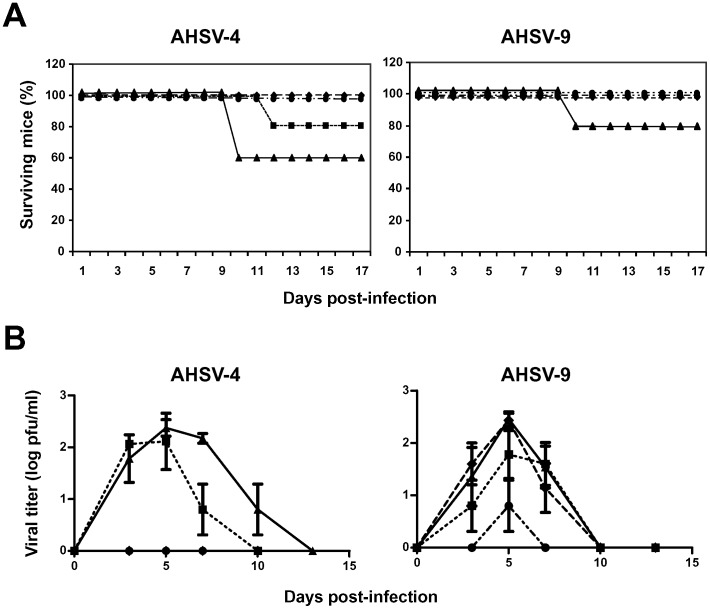
Protection of rMVA/rMVA vaccinated IFNAR^(−/−)^ mice against an AHSV-4 or AHSV-9 challenge. Mice (8 weeks old, 6 per group) were immunized twice by homologous prime-boost vaccination with rMVA expressing VP2 (**♦**), NS1 (▪), or VP2/NS1 (•), AHSV-4 proteins or MVA **(**▴**)** (non-immunized mice), administered 3 weeks apart. Two weeks after immunization all mice were subcutaneously inoculated with 10^6^ PFUs of AHSV-4 or AHSV-9. (A) Survival rates of immunized and non-immunized IFNAR^(−/−)^ mice after inoculation with AHSV-4 or AHSV-9. The mice were observed every 24 h for 17 days. (B) Titers of AHSV-4 or AHSV-9 recovered in blood of immunized and non-immunized IFNAR^(−/−)^ mice after challenge. Virus was extracted from blood and determined as described in Materials and Methods. Each point represents the mean values of the viral titer of six animals, and standard deviations are shown as error bars.

In order to improve the protection conferred by the antigens VP2 and NS1 against homologous and heterologous challenge with AHSV, adult IFNAR^(−/−)^ mice were immunized twice with rMVA-VP2 and/or rMVA-NS1 by intraperitoneal injection three weeks apart. Previous studies demonstrated that two immunizations with rMVA expressing VP2 protected IFNAR^(−/−)^ mice against AHSV-4 [12]. Two weeks after the vaccination boost, immunized and control IFNAR^(−/−)^ mice were challenged subcutaneously with 10^6^ PFUs of AHSV-4 or 10^6^ PFUs of AHSV-9. The percentage survival showed that all the mice vaccinated with VP2 or VP2/NS1 survived the challenge with AHSV-4 (Fig. 3A). In contrast, the non-immunized and the NS1 immunized groups presented a survival rate of 60% and 80%, respectively. All of the rMVA-VP2 and rMVA-VP2/NS1 vaccinated animals were also protected from clinical signs and were completely healthy until the end of the study. In contrast, all the non-vaccinated control mice and the animals immunized with rMVA-NS1 developed clinical signs similar to those described earlier during the previous challenge experiment. Non-immunized mice infected with AHSV-9 presented a milder clinical syndrome characterised by reduction of mobility and eye swelling. In contrast, the groups of animals immunized with NS1, VP2 or both antigens did not show clinical signs after challenge.

Viraemia after immunization and challenge was determined by virus isolation on cell culture from whole blood. Non-immunized and NS1 immunized animals infected with AHSV-4 showed titers up to 4.4×10^2^ PFU/ml and 3.5×10^2^ PFU/ml, respectively, at 5 days post-challenge. No viraemia was detected in the animals immunized with VP2 or VP2/NS1. After an heterologous challenge with AHSV-9, titers up to 3.4×10^2^ PFU/ml, 4.4×10^2^ PFU/ml, and 2.8×10^2^ PFU/ml were detected at 5 days post-challenge in non-immunized, VP2, and NS1 immunized mice, respectively. Lower titers up to 80 PFU/ml were detected in the animals immunized with VP2/NS1 but only at day 5 post-challenge with AHSV-9 (Fig. 3B).

These data indicate that the immunization of mice with rMVA-VP2 or rMVA-VP2/NS1 induces total protection against an homologous challenge with AHSV-4. Furthermore, VP2/NS1 of AHSV-4 induced protection against an heterologous challenge with AHSV-9 and reduced the viraemia almost completely.

### Homologous and Heterologous Prime-boost Immunization with DNA or rMVA Expressing VP2 from AHSV-4 Elicit Neutralizing Antibodies against AHSV-4 but not against AHSV-9 in IFNAR^(−/−)^ Mice

**Figure 4:**
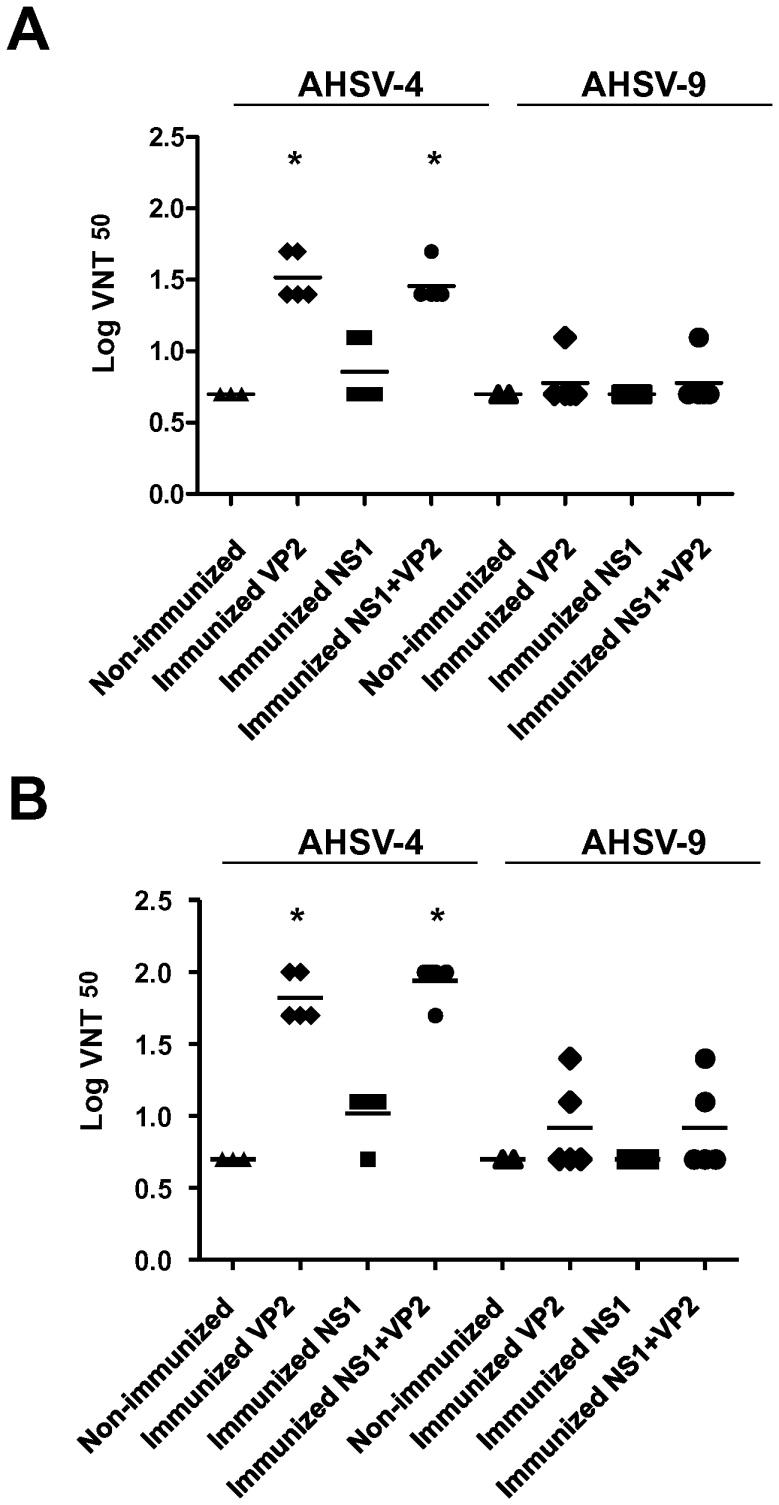
Humoral immune response observed in IFNAR^(−/−)^ mice vaccinated with DNA/rMVA or rMVA/rMVA expressing VP2/NS1. Neutralizing antibodies specific of AHSV-4 or AHSV-9 were analyzed in sera of immunized mice by VNT. Neutralization titers at day 15 post-booster treatment in sera of animals immunized with DNA/rMVA (A) or rMVA/rMVA (B) expressing VP2, NS1, or VP2/NS1 are shown. (▴) Non-Immunized, (♦) VP2 immunized, (▪) NS1 immunized, and (•) VP2/NS1 immunized. Means are presented as bars (⁃). Asterisks (*) indicate statistically significant differences (*P*<0.05) between immunized and non-immunized mice, calculated by signed rank test.

The presence of serotype-specific neutralizing antibodies against AHSV-4 and AHSV-9 in the sera of DNA/rMVA or rMVA immunized mice was analyzed by virus neutralization tests (VNT) before challenge. Neutralizing antibodies against AHSV-4 were observed in mice immunized with DNA/rMVA-VP2 or DNA/rMVA-VP2/NS1 two weeks after booster treatment with rMVAs with a Log VNT^50^ of 1.51 and 1.45 respectively (Fig. 4A). The levels of neutralizing antibodies detected in mice immunized with DNA/rMVA-NS1 were similar to those measured in non-immunized mice. In contrast, neutralizing antibodies against heterologous AHSV-9 were not observed in the serum of immunized or non-immunized mice (VNT^50^≤0.3) at the analyzed time. Mice vaccinated with rMVA-VP2 or rMVA-VP2/NS1 developed neutralizing antibodies against AHSV-4 after the second dose of vaccine was given with a Log VNT^50^ of 1.81 and 1.93, respectively (Fig. 4B). In contrast, comparison of VNT^50^ specific of AHSV-9 between serum from vaccinated and non-vaccinated mice did not show statistical significance by the Student's t-test (p<0.05). In addition, neutralizing antibodies against AHSV-4 and AHSV-9 were not detected in sera from non-immunized mice and DNA/rMVA or rMVA immunized mice at 14 days post-immunization (data not shown).

The lack of or poor neutralizing antibody response to serotype 9 in vaccinated mice indicates that VP2 and NS1 from AHSV-4 do not induce detectable cross-reactive neutralizing antibodies against serotype 9.

### Heterologous Prime-boost Vaccination with pcDNA3-VP2/pcDNA3-NS1 and rMVA-VP2/rMVA-NS1 Induces the Generation of Specific T cell Responses

**Figure 5:**
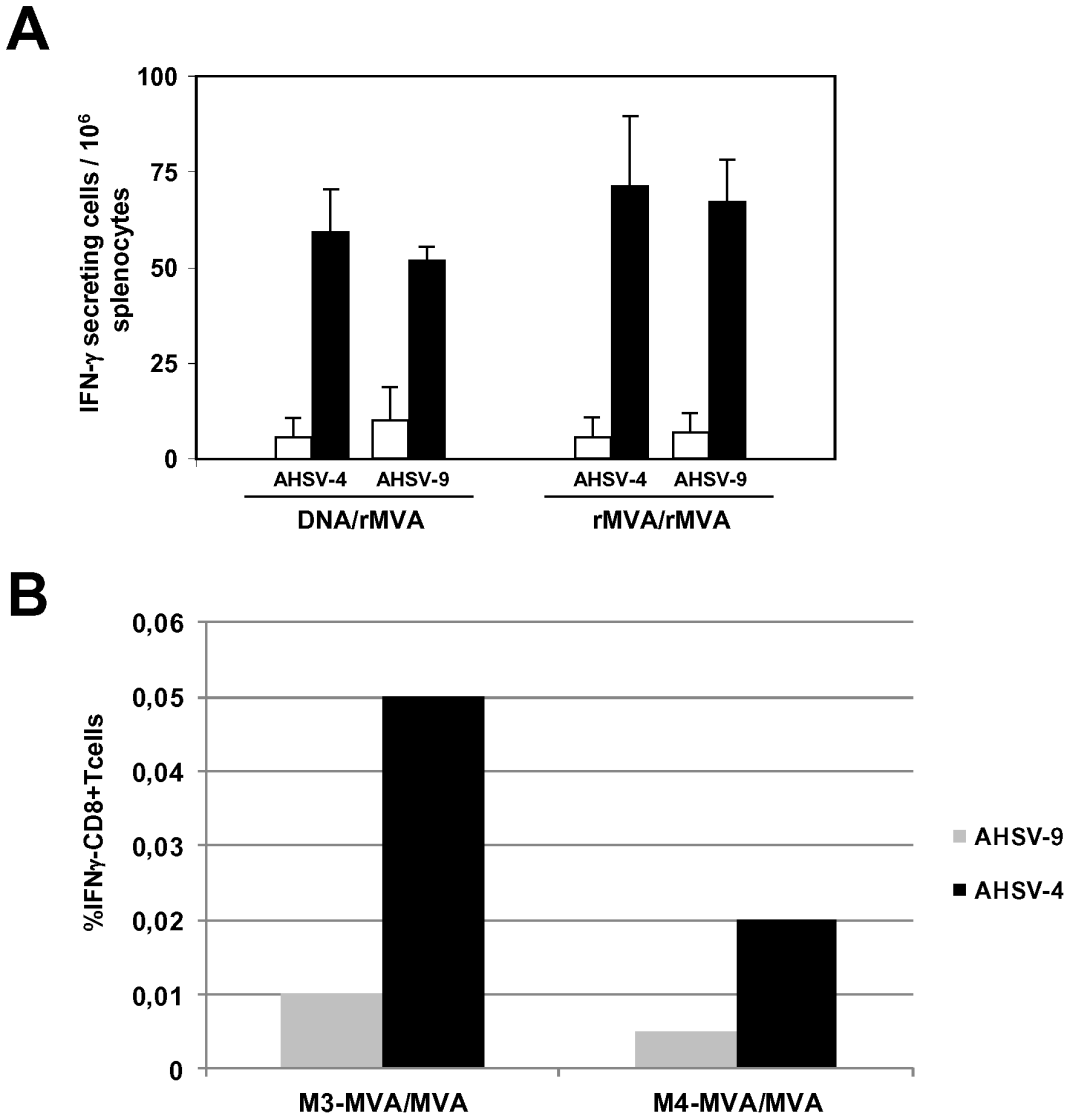
Cellular immune response observed in IFNAR^(−/−)^ mice vaccinated with DNA/rMVA or rMVA/rMVA expressing VP2/NS1. (A) ELISPOT assays measuring IFN-γ-secreting T cells in the spleen of immunized mice. Mice were immunized with DNA/rMVA or rMVA/rMVA expressing VP2 and NS1 from AHSV-4 as described in Materials and Methods. Splenocytes were harvested at day 14 post-vaccination. Non-immunized mice were used as controls. Black (immunized) and white (non-immunized) bars represent the SFC mean number ± standard deviation for the ELISPOT within each group. 10^4^ PFUs of UV inactivated virus per well were used as stimulus in each experiment. (B) Intracellular staining of IFN-γ, in T CD8+ cells of rMVA/rMVA-VP2/NS1 immunized IFNAR(−/−) mice. Two weeks after second immunization, spleens were harvested and the splenocytes were stimulated with 10^4^ PFUs of UV inactivated virus per well. At 24 h post-stimulation, intracellular IFN-γ production was analysed in CD8-positive cells by flow cytometry. Grey bars: mice stimulated with AHSV-9; black bars: mice stimulated with AHSV-4. M3-MVA/MVA and M4-MVA/MVA are the values of two representative rMVA/rMVA immunized mice.

To further analyze the protective immune response elicited by the DNA/rMVA and rMVA/rMVA vaccines, the amount of IFN-γ-producing spleen cells after the immunizations was determined by ELISPOT. IFNAR^(−/−)^ mice were immunized by homologous or heterologous prime-boost vaccination with DNAs and rMVAs expressing VP2 and NS1 proteins from AHSV-4 or DNA and MVA (controls), administered 3 weeks apart. Two weeks after second immunization spleens were harvested and the splenocytes were stimulated with UV inactivated AHSV-4 or AHSV-9 in ELISPOT plates. As shown in Figure 5A, mice immunized with both strategies, DNA/rMVA and rMVA/rMVA, developed detectable specific IFN-γ producing cells after stimulation with UV inactivated AHSV-4 and AHSV-9 when compared to the non-immunized group. The mean value of IFN-γ producing cells after stimulation with AHSV-4 was higher than after stimulation with AHSV-9, although the difference was not significant when the results were analyzed by Students t-test. In addition, higher mean value of IFN-γ producing cells was observed in mice immunized with rMVA/rMVA than in the DNA/rMVA immunized mice, but the difference was not significant either.

To analyze the phenotype of the AHSV-specific IFN-γ producing cells induced *in vivo*, intracellular cytokine staining was performed. Whole splenocytes of DNA/rMVA-VP2/NS1 or rMVA/rMVA-VP2/NS1 immunized mice were re-stimulated with UV inactivated AHSV-4 or AHSV-9 for 24 h and intracellular IFNγ production by CD8+ T and CD4+ T cells was then determined by flow cytometry upon treatment of the cells with the golgi inhibitor, brefeldin A. UV inactivated AHSV-4 and AHSV-9 did not induce the expression of IFNγ by CD4+ T cells upon re-stimulation in rMVA/rMVA-VP2/NS1 or DNA/rMVA-VP2/NS1 immunized mice (data not shown). In contrast, CD8+ T cells were only stimulated in splenocytes of the animals immunized with rMVA/rMVA-VP2/NS1 after stimulation with both UV inactivated AHSV (Fig. 5B), although the level of stimulation was higher with AHSV-4 than with AHSV-9. These data suggest that although the two strategies of immunization assayed induced an immune cellular response in the mice, only the animals immunized with rMVA/rMVA-VP2/NS1 elicited an evident CD8+ T response that could be essential for a complete protection against multiple serotypes of AHSV.

## Discussion

Recent outbreaks of bluetongue in Europe (particularly the northern European outbreak caused by BTV-8) have clearly demonstrated an increasing threat to animals posed by bluetongue virus (BTV), and consequently by other related orbiviruses that are transmitted by the same biting midge vectors such as: African horse sickness virus (AHSV), Equine Encephalosis virus (EEV), and Epizootic Haemorrhagic disease virus (EHDV). Consequently, the risk of African horse sickness (AHS) outbreaks may increase in areas traditionally free of the disease, including the Middle East, North Africa and Europe and perhaps other parts of the world [16].

A variety of vaccines have been developed to prevent AHSV infection of equids. The major antigenic capsid protein ‘VP2’, either on its own, or co-expressed with VP5, VP3, and VP7 to form virus-like-particles (VLPs), induced virus neutralizing antibodies and protective immunity against AHSV [28], [29]. Other AHSV vaccination strategies have been based on recombinant live viral vectors, specially the poxviruses. Thus, a recombinant wild-type vaccinia virus expressing VP2 of AHSV-4 was shown to protect horses against virulent challenge [13]. Similarly, MVA was also an effective vaccine-vector for AHSV. Good protein expression was demonstrated in equine skin fibroblast or avian cells infected with recombinant MVA viruses encoding AHSV VP2, VP7, or NS3. Furthermore, vaccination of ponies with recombinant MVA induced an AHSV-specific immune response that (in the case of rMVA-VP2) included development of neutralizing antibodies [11]. In addition, rMVA-VP2 was tested in IFNAR^(−/−)^ mice and the immunization induced sterile protection in the vaccinated animals [12]. Similarly, recombinant canarypox virus, expressing both major outer-capsid antigens, VP2 and VP5, induced protective immunity [16], [30].

In this work we have developed recombinant AHSV-vaccines that are inherently safer and compatible with a DIVA approach. First, the efficacy of a homotypic vaccination with MVA vectors, and a heterotypic vaccination with DNA and MVA vectors expressing VP2 and NS1 proteins from AHSV-4 has been assayed. IFNAR^(−/−)^ mice, a small animal model of BTV, AHSV, and EHDV infection [12], [20], [31], have been used in the present work in order to study the protection and immune response conferred by the homologous and heterologous prime-boost vaccination with DNA and rMVA expressing VP2 and NS1 from AHSV-4. Protection indicators used in our studies included primarily the observation of clinical signs and viraemia of challenged mice. Non-immunized mice infected with AHSV-4 or AHSV-9 showed similar clinical signs (rough hair coat, a hunched posture, reduction of mobility, and lacrimation and swelling of the eyelids), but in the case of the animals infected with serotype 9, these clinical signs were milder and the mortality rate was very low. In contrast, the level of viraemia was similar in the animals infected with the serotypes 4 or 9, although the period of viraemia was shorter in mice infected with AHSV-9. For this reason, in this study, viraemia has been the main protection indicator to analyze the potency of the vaccination strategies and the best vaccine composition to induce cross-protection.

Immunization with DNA/rMVA or rMVA/rMVA expressing VP2 from AHSV-4 protected the mice against an homologous challenge with AHSV-4. Mice immunized with DNA/rMVA showed a reduction of viraemia and the animals immunized with rMVA/rMVA did not present infective virus in blood. This observation indicates that the strategy based on an homologous prime-boost with rMVA expressing AHSV proteins is more effective against the AHSV infection than the heterologous prime-boost with DNA/rMVA. Previous work in our laboratory showed that the inclusion of NS1 in the composition of BTV vaccines cross-protected against the challenge with heterologous serotypes of this virus [19]. In the case of AHSV, when NS1 was included in combination with VP2 from AHSV-4 in the vaccine composition, both strategies cross-protected against an heterologous challenge with AHSV-9. We observed that the virulence of AHSV-9 was lower than the infection with AHSV-4 in the IFNAR^(−/−)^ mice model with a shorter period of viraemia and mild clinical signs. This lower virulence of the AHSV-9 infection in the IFNAR^(−/−)^ mice model would explain that the efficacy of both strategies that express VP2 and NS1 proteins from AHSV-4 was similar in the heterologous challenge.

The efficiency at generating neutralizing antibodies has been described as a surrogate marker of protection in the AHSV vaccines. Immunization with DNA/rMVA or rMVA/rMVA expressing VP2 alone or in combination with NS1 from AHSV-4 induced neutralizing antibodies specific of serotype 4. The serological cross-reactivity between certain AHSV serotypes has been described. Cross-reactivity between serotypes 1 and 2, serotypes 3 and 7, serotypes 5 and 8, and serotypes 6 and 9, has been reported, while serotype 4 does not cross react with any other serotype [32]. Interestingly, we observed that heterologous neutralizing antibodies to serotype 9 were not detected in mice immunized with DNA/rMVA or rMVA/rMVA expressing AHSV-4 VP2 alone or in combination with AHSV-4 NS1. However, these mice were protected against AHSV-9. Previous studies reported that horses vaccinated with purified VP2 and VP7 of AHSV-4 or with the recombinant canarypox *ALVAC*-AHSV-4 expressing VP2 and VP5 did not seroconvert but were protected against virulent challenge with AHSV-4 [16], [29]. These studies suggested that cell-mediated immune mechanisms were playing a role in the protection of these vaccinated animals. Studies performed with BTV, a related orbivirus, demonstrated that VP2 and NS1 are major CTL immunogens in sheep [17], [18]. In addition, we showed that vaccination of IFNAR^(−/−)^ mice with DNA/rMVA expressing VP2, VP7 and NS1 of BTV-4 achieved protective heterotypic immunity and protection against heterologous infection with BTV-8 and BTV-1 by inducing a strong T cell immune response [19]. The ELISPOT results showed that mice immunized with DNA/rMVA or rMVA/rMVA expressing VP2 and NS1 from AHSV-4 elicited specific IFN-γ responses. UV inactivated AHSV-4 or AHSV-9 used as stimuli resulted in significant IFN-γ responses by splenocytes of immunized mice, especially the rMVA/rMVA immunized mice, strategy that conferred more efficient protection against the virus. This data and the fact that not significant differences in IFN-γ responses were observed between stimulation with AHSV-4 and AHSV-9 confirm the importance of cell mediated immunity in the induction of homotypic and heterotypic protection against AHSV infection.

Protective immunization of horses with an attenuated AHSV-4 or a recombinant canarypox virus vectored vaccine co-expressing VP2 and VP5 induced an increase of CD8+ cells able to recognize multiple T-epitopes in the AHSV proteins [16], [30], [33] **.** The proliferation of virus-specific CD8+ T cells suggests that these T-cells may play a role in protective immunity. The ELISPOT results detected the stimulation of a number of activated cell populations that secrete IFN-γin the presence of inactivated virus. These can include NK-cells, alpha/beta TCR and gamma/delta TCR expressing T-cells as previous reports have described [30]. Furthermore, ICCS assays showed the presence of AHSV-specific CD8+ T cells in mice immunized with rMVA/rMVA expressing VP2 and NS1 but not in mice immunized with DNA/rMVA, data that could explain the difference of efficacy between both strategies of vaccination. Furthermore, CD8+ T cells of mice immunized with rMVA/rMVA-VP2/NS1 were stimulated with both serotypes of AHSV, serotypes 4 and 9, confirming the importance of including NS1 in the composition of a multiserotype vaccine against AHSV.

In summary, two strategies of vaccination based on homologous or heterologous prime-boost of naked DNA and rMVA expressing VP2 and NS1 proteins of AHSV-4 have been assayed against the infection with serotypes 4 or 9 of AHSV. The immunization with rMVA/rMVA was more efficient in protection against a virulent challenge with AHSV-4 and both strategies, DNA/rMVA and rMVA/rMVA, protected against the infection with AHSV-9, serotype with a lower virulent behavior in the infections of IFNAR^(−/−)^ mice. The inclusion of NS1 in the vaccine composition, as previously was described for BTV vaccines [19], elicits cross-protection against heterologous infections of AHSV. NS1 protein of orbiviruses has been reported to be conserved among serotypes and its inclusion in the composition of recombinant marker vaccines could be important to generate effective protective orbivirus multiserotype vaccines.

## Ackowledgments
